# Practice of integrated vector surveillance of arthropod vectors, pathogens and reservoir hosts to monitor the occurrence of tropical vector-borne diseases in 2020 in Zhejiang Province, China

**DOI:** 10.3389/fvets.2022.1003550

**Published:** 2022-11-16

**Authors:** Yuyan Wu, Jinna Wang, Qinmei Liu, Tianqi Li, Mingyu Luo, Zhenyu Gong

**Affiliations:** Department of Infectious Diseases Control and Prevention, Zhejiang Provincial Center for Disease Control and Prevention, Hangzhou, China

**Keywords:** integrated vector surveillance system, vector-borne diseases, monitoring, ecology surveillance, etiology surveillance, insecticide sensitivity test

## Abstract

**Background:**

Vector-borne diseases have become one of the most serious local public health threats. Monitoring and controlling vectors are important means of controlling vector-borne diseases. However, traditional vector surveillance systems in China mainly monitor vector density, making its early-warning effect on vector-borne diseases weak. In this study, we applied an integrated surveillance system of multiple arthropod vectors and reservoir host containing ecology, etiology, and drug resistance monitoring to obtain better knowledge on vector populations and provide early warning of suspicious vector-borne infectious disease occurrence.

**Methods:**

An ecology surveillance of mosquitoes, rodents, ticks, and chigger mites, a pathogen infection survey on mosquitoes and rodents, and a drug resistance survey on *Aedes albopictus* were conducted in 12 cities in Zhejiang Province in 2020.

**Results:**

A total of 15,645 adult mosquitoes were collected at a density of 19.8 mosquitoes per Centers for Disease Control and Prevention light trap. *Culex tritaeniorhynchus* (72.76%) was the most abundant species. The Breteau index of *Ae. albopictus* was 13.11. The rodent density was 0.91 rodents per hundred traps; the most abundant species was *Rattus norvegicus* (33.73%). The densities of dissociate and ectoparasitic ticks were 0.79 ticks per hundred meters and 0.97 ticks per animal, respectively. The most abundant tick species was *Haemaphysalis longicornis* (56.38%). The density of chigger mites was 14.11 per rodent; two species were identified, with the most abundant species being *Walchia* spp. mite (68.35%). No flavivirus or alphavirus was found in mosquito etiology monitoring, whereas the positivity rates of hantavirus, the pathogenic bacteria *Leptospira* spp., *Orientia tsutsugamushi*, and *Bartonella* spp. detected in rodent etiology monitoring were 1.86, 7.36, 0.35 and 7.05%, respectively. Field populations of *Ae. albopictus* in Zhejiang Province were widely resistant to pyrethroids but sensitive to most insecticides tested, including organophosphorus and carbamate insecticides.

**Conclusion:**

Integrated surveillance systems on multiple arthropod vectors (mosquitoes, ticks, mites) and animal reservoirs (rodents) can provide important information for the prevention and control of epidemic emergencies.

## Introduction

Arthropod-vector-borne diseases (VBDs) include many widespread diseases, such as malaria, yellow fever, dengue fever, plague, and Lyme disease. Moreover, 80% of the global population is at risk of ≥1 VBDs, and >700,000 people die from VBDs annually (1). The burden of VBDs accounts for 17% of the global infectious disease burden (1). Consequently, VBDs have become one of the most serious global public health threats (2, 3).

In China, with the risk of global warming, changes in the ecological environment, increased trade, and population mobility, epidemics of VBDs have become increasingly serious (4, 5). Mosquito-borne diseases (MBDs) can be considered as an example. Because of warm temperature, the distribution range of *Aedes albopictus* in China continues to expand, leading to ≥168 million people being at high risk of dengue fever annually (3, 6–10). In 2019, 22,188 confirmed dengue fever cases were reported in mainland China by the National Notifiable Disease Reporting System, causing huge economic losses (11). Without effective medicine and vaccines, monitoring and controlling arthropod vectors and reservoir hosts have been important means of controlling VBDs.

Monitoring systems for reservoir hosts, vectors, and pathogens are important early-warning systems to prevent the occurrence or reduce the severity and duration of VBD outbreaks (12). Recently, different types of monitoring systems have been established worldwide, playing important roles in preventing VBDs (12–16). For example, the West Nile virus annually causes multiple outbreaks associated with human and equine mortality in European countries, and targeted surveillance for mosquito populations and pathogens within them has been regarded as a cost-effective way to manage this threat (17). Early detection was supposed to offer opportunities to raise diseases awareness, initiate vector control, and encourage personal protection (17). However, in China, the aim of traditional vector surveillance is mainly to monitor the density of traditional vectors, such as mosquitoes, flies, rodents, and cockroaches. Without information about vector-borne pathogen activity in vectors, an early warning system is insufficient. Thus, in this study, we enforced an integrated surveillance system of multiple arthropod vectors (mosquitoes, ticks, and chiggers) and reservoir hosts (rodents) in the Zhejiang Province.

Zhejiang Province is located on the southeast coast of China and has a subtropical monsoon climate. A suitable climate and developed tourism have made Zhejiang one of the provinces with the highest incidence rate of VBDs in China. From 2015 to 2019, there has been an annual epidemic of dengue fever, and >95% of the counties in Zhejiang have reported dengue fever cases (18). Huge pressure to prevent VBDs forced Zhejiang to try various monitoring methods to prevent related diseases. For the integrated surveillance of multiple arthropod vectors and reservoir, the ecology, etiology, and drug resistance of targeted vectors were monitored to determine the prevalence of vector-borne pathogens in vectors and their density to predict the possibility of related VBDs becoming prevalent in the population.

In this study, we analyzed the density and constitution of targeted vectors and reservoir hosts, identified potential vectors of vector-borne pathogens, and investigated pathogen infection among vectors and reservoir host in an integrated system in 2020 to gain more knowledge on vector populations and provide early warning of suspicious VBDs occurrence.

## Materials and methods

### Ethics statement

No permission was required for the described studies. These studies did not involve endangered or protected species; mosquito and rodent collection for monitoring was provided by the owners at each location.

### Study design

The 2020 integrated surveillance in Zhejiang Province comprised vector surveillance, pathogen surveys, and resistance surveys, with the aim of monitoring the ecology, etiology, and drug resistance of targeted vectors and hosts containing mosquitoes, rodents, ticks, and chigger mites. Twelve cities (Hangzhou, Ningbo, Wenzhou, Huzhou, Jiaxing, Shaoxing, Jinhua, Quzhou, Lishui, Taizhou, Zhoushan, and Yiwu) in Zhejiang Province formed the study area, comprising 105,500 km^2^, with approximately 64.6 million habitants.

### Vectors and reservoir hosts surveillance

**Figure 1:**
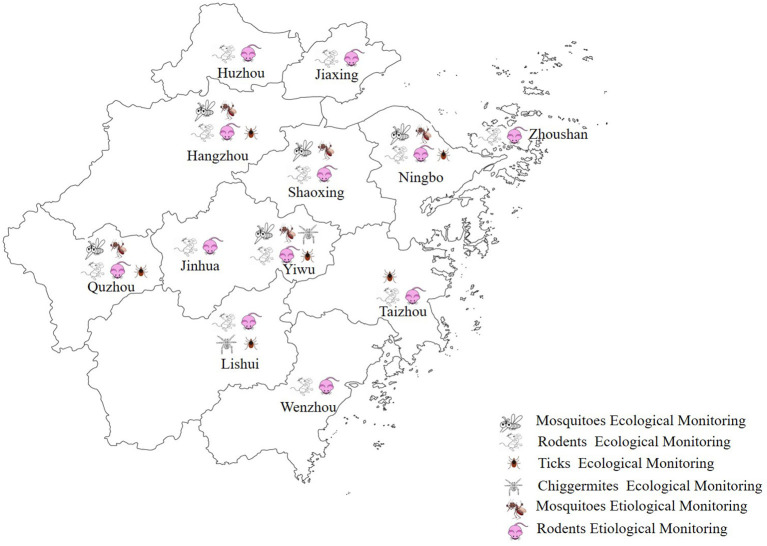
A map of Zhejiang Province showing the locations of the 12 monitoring sites and the tasks conducted at these sites in the integrated surveillance system in 2020.

The ecology of mosquitoes, rodents, ticks, and chigger mites was monitored in 2020; details of their locations are shown in Figure 1.

#### Mosquito surveillance

Mosquitoes were monitored from July to November, with monthly collections of adult and larval mosquitoes. Adult mosquitoes were trapped using Centers for Disease Control and Prevention (CDC) light traps in farmhouses, livestock sheds, urban residential quarters, and gardens at each monitoring site per month. Larvae mosquitoes were monitored using the larval pipette method recommended by the National Standards of the People's Republic of China (GB/T 23797–2009) in ≥100 residential areas at each monitoring site per month, using which the Breteau index (BI) was calculated for *Ae*. albopictus (19).

#### Rodent surveillance

Rodents were monitored in a single month (July, September, and November) in urban resident quarters, high-risk industries (i.e., restaurants and slaughterhouses), and rural farmhouses in each monitoring site per month using ≥600 rat traps with the night trapping method recommended by the National Standards of the People's Republic of China (GB/T 23798–2009) (20).

#### Tick surveillance

Ticks were monitored in July and September, with monthly collection of ectoparasitic and dissociate ticks. Ectoparasitic ticks were monitored in urban and rural animals, such as livestock and pets, and dissociate ticks were collected from barren grasslands in rural countries with a 90 × 60-cm white fleece dragging on the grass floor.

#### Chigger surveillance

Chigger mites were monitored in July and September, in combination with rodent surveillance. If the number of rodents caught in rodent surveillance was <30 rodents, additional rodents were caught in each chigger mite monitoring site. After rodents were sampled, the chigger mites on their surfaces were collected.

All vectors collected were sent to local municipal CDC maintaining the cold chains to identify the species.

### Pathogen surveillance

Etiological surveillance for mosquitoes and rodents was conducted in this study; details on the collection sites are shown in Figure 1. At every mosquito etiological monitoring site, ≥2,500 mosquitoes, including 1,000 *Ae. albopictus*, 1,000 *Culex tritaeniorhynchus*, and 500 other species were sampled. All sampled mosquitoes were pooled according to species, habitat, and date, with a maximum of 30 individuals per pool. RNA/DNA was extracted using the Magnetic Viral DNA/RNA Fast Kit (TIANGEN, China) according to manufacturer's instructions and stored at −80 °C. RNA was reverse transcribed using the PrimeScript^TM^ One Step RT-PCR Kit version 2 (Takara, Japan). After the first amplification round, the product was used as a template for the second round of amplification using Takara Ex Taq^TM^ version 2.0 plus dye (Takara, Japan). The primer concentrations used in PCR reactions were 0.2 μmol/μL, and the polymerase chain reaction (PCR) amplification was performed as follows: denaturation at 95 °C for 2 min, followed by 30 cycles at 95 °C for 1 min, annealing at 72 °C for 10 s, and final extension step at 72 °C for 5 min. Primer sequences, amplification fragments, and other information are listed in Table 1. To ensure the reliability of the experimental results, positive (Dengue virus type II for flavivirus and Chikungunya virus for alphavirus, provided by China CDC), negative (double-distilled water, ddH2O) and blank controls (all PCR reaction chemicals except the template DNA) were used for each experiment. The PCR products were analyzed using 1.2% agarose gel (GelREd Nucelic Acid Gel Stain, 10000X, Biontium) electrophoresis ultraviolet imaging using Shanghai QIN XIANG Gel imager (model: GenoSens 2200).

**Table 1:** Primers for testing *flavivirus* and *alphavirus* in mosquitoes using reverse transcription-hemi-nested polymerase chain reaction amplification.

**Pathogens**	**PCR amplification**	**Primers**	**Primer sequences(5^′^~3^′^)**	**Annealing temperature (°C)**	**Fragment size (bp)**
Flavivirus	Round 1	XF-F1	AACATGATGGGVAARMGWGARAA	52	263
		XF-R	GTRTCCCANCCDGCDGTRTCATCNGC		
	Round 2	XF-F2	AARGGMAGYMGNGCHATHTGGT	56	215
		XF-R	GTRTCCCANCCDGCDGTRTCATCNGC		
Alphavirus	Round 1	XA-F1	AGAGCRTTYTCGCATCTRGCYAK	51	433
		XA-R	ACATGAACKGRGTKGTGTCRAASCCWAYCC		
	Round 2	XA-F2	TGCCCBRTGCGBAGYSCVGAAGAYCC	61	310
		XA-R	ACATGAACKGRGTKGTGTCRAASCCWAYCC		

At every rodent etiological monitoring site, ≥100 rodents were sampled and rodent organs, including the liver, spleen, lung, and kidney were removed from all sampled rodents to test for two bacterial pathogens among the pathogenic bacteria *Leptospira* spp., *Rickettsia mordeni, Anaplasma phagocytophilum, Bartonella* spp., *Orientia tsutsugamushi, Yersinia* spp., and *Francisella tularensis*, and two viral pathogens, hantavirus and severe fever with thrombocytopenia syndrome bunia virus (SFTSV). Total nucleic acids (including DNA and RNA) were extracted using a Magnetic Viral DNA/RNA Fast Kit (TIANGEN, China) and amplified by real-time fluorescence quantitative PCR assay for target genes (shown in Table 2). For the pathogenic bacteria *Leptospira* spp., *R. mordeni, A. phagocytophilum, Bartonella* spp., *O. tsutsugamushi*, and *F. tularensis*, 20 μL of a Takara PrimeScript^TM^ one step PT-PCR kit (Takara, Japan) was used; cycling parameters included one cycle of denaturation at 95 °C for 5 min, followed by 40 cycles of amplification at 95 °C for 15 s and 60 °C for 45 s. Cycle threshold values <35 were considered positive. Real-time PCR assays for *Yersinia* spp., hantavirus and SFTSV were performed according to Wei et al., Pang et al. and Sun et al. (21–23). Positive standards for target pathogens were provided by China CDC.

**Table 2:** Primers and probes for testing bacterial and viral pathogens in rodents.

**Pathogens**	**Primers and probes(5'-3')**	**Seguences**	**Concentrations in reactions (μmol/μL)**
*Rickettsia mordeni*	Pr47F	TGTTGATGGTGCAGGATTTGA	0.25
	Pr110R	CGAATTTGTAGCGACAGGAAGA	0.25
	mo-T	FAM-CAAACTGGCGCTGGTGT-MGB	0.15
*Anaplasma phagocytophilum*	wu-FP	CCACGCAAGTCGCATTGAT	0.25
	Wu-RP	GCCGGGTACTTTCGCAATT	0.25
	Wu-T	FAM-CTTACAGGTGCTATCATC-MGB	0.15
*Orientia tsutsugamushi*	Ot56 kD-F	CGCCAGTRATMATTCCTCCRA	0.25
	Ot56 kD-R	TTTYWGCTAGTGCRATAGAATTRG	0.25
	Taqman-Ot	FAM-TAAGGACCACACTCTAATC-MGB	0.15
*Francisella tularensis*	AKR-F	GCAGGGCGAGCACCATT	0.25
	AKR-R	ATCTTGCATGGTCACCACTTGA	0.25
	AKR-T	FAM-CGATATTTGCCTGTTAGCACTCCT-BHQ	0.15
*Bartonella* spp.	ssrA-F	GCTATGGTAATAAATGGACAATGAAATAA	0.25
	ssrA-R	GCTTCTGTTGCCAGGTG	0.25
	ssrA-T	FAM-ACCCCGCTTAAACCTGCGACG-BHQ1	0.15
The pathogenic bacteria *Leptospira* spp.	Lepto F	CCCGCGTCCGATTAG	0.25
	Lepto R	TCCATTGTGGCCGRAACAC	0.25
	Lepto P	FAM-CTCACCAAGGCGACGATCGGTAGC-BHQ	0.15

### Insecticide resistance surveillance in *Ae. Albopictus*

Field populations from five monitoring sites were collected during the 2020 active mosquito season in Hangzhou, Ningbo, Shaoxing, Quzhou, and Yiwu, China. Female *Ae. albopictus* mosquitoes were tested for susceptibility to four pyrethroid insecticides (0.07% lambda-cyhalothrin, 0.08% beta-cypermethrin, 0.03% deltamethrin, and 0.4% permethrin), three organophosphorus insecticides (0.5% malathion, 0.2% fenitrothion, and 2% chlorpyrifos), and two carbamate insecticides (0.05% propoxur and 0.2% bendiocarb) using the World Health Organization (WHO) bioassay according to the test protocol recommended by the China CDC (24, 25). The 24 h mortality rate was calculated, and resistance status was classified according to WHO recommendations: susceptibility, if the 24 h mortality rate was 98–100%; probable resistance, if the 24 h mortality rate was 90–97%; and resistance, if the 24 h mortality rate was <90%. Mosquitoes were considered dead if they were motionless.

### Statistical analyses

Data preparation and statistical analyses were performed using the Statistical Package for the Social Sciences version 23.0. A descriptive analysis was performed to display the vector population in the ecological monitoring system. Flavivirus and alphavirus infection prevalence in mosquitoes was estimated using pooled samples. Bacterial and viral pathogen infections in rodents were determined in the tested samples.

## Results

### Arthropod vectors and reservoir hosts surveillance

A total of 15,645 adult mosquitoes were collected by 790 CDC light trap stations from all four monitoring sites, with a density of 19.8 mosquitoes per trap. Yiwu had the highest density whereas Hangzhou had the lowest density (Table 3). Four species were identified; *Cx. tritaeniorhynchus* as the most abundant species (72.76%), collected mainly in Yiwu, followed by *Cx. pipiens pallens* (10.41%) and *Ae. albopictus* (1.26%), which were captured in all four sites. A total of 5,798 residential areas were visited; *Ae. albopictus* larvae were found in 760 containers, with a BI of 13.11. Yiwu had the highest BI (28.20), whereas Hangzhou had the lowest BI (7.53).

**Table 3:** Arthropod vectors and reservoir hosts collected in different monitoring sites in 2020 in Zhejiang Province, China.

**Monitoring site**	**Rodents**		**Mosquitoes**		**Ticks**		**Chigger mites**	
	**No. of traps**	**No. of rodents (%)**	Adult density^*^	Larval BI ^**^	**Ectoparasitic ticks density ^※^**	**Dissociate ticks density^※※^**	**No. of Chigger mites**	**Chigger index^&^**
Hangzhou	2446	3 (0.12)	1.22	7.53	0.00	0.00	/	/
Ningbo	2495	2 (0.12)	6.07	24.96	0.64	0.43	/	/
Quzhou	2445	24 (0.98)	1.65	8.16	0.00	0.00	/	/
Yiwu	426	12 (2.82)	133.13	28.20	1.33	0.00	423	13.22
Lishui	1200	7 (0.58)	/	/	1.12	4.45	480	15.00
Taizhou	2534	8 (0.32)	/	/	6.60	0.00	/	/
Shaoxing	10256	79 (0.77)	/	/	/	/	/	/
Jiaxing	2457	7 (0.28)	/	/	/	/	/	/
Jinhua	2457	30 (1.22)	/	/	/	/	/	/
Wenzhou	2201	76 (3.45)	/	/	/	/	/	/
Zhoushan	3671	78 (2.12)	/	/	/	/	/	/
Huzhou	3709	6 (0.16)	/	/	/	/	/	/
Total	36297	332 (0.91)	19.8	13.11	0.97	0.79	903	14.11

* Adult density, number of adult mosquitoes with the number of traps.

** Larval BI, number of containers housed with Aedes albopictus larvae/number of households inspected × 100.

※ Ectoparasitic tick density, number of ticks caught in animals/number of animals tested.

※※ Dissociate tick density, number of ticks caught/distance of dragging white fleece × 100 m.

& Chigger index, number of chigger mites caught/number of rodents tested.

A total of 36,297 rat traps were set at 12 monitoring sites; 332 rodents were caught in 2020 at a rate of 0.91% (Table 3). Fifteen species were identified; the most abundant being *Rattus norvegicus* (33.73%), followed by *Mus musculus* (17.47%), *Rattus flavipectus* (17.17%), *Suncus murinus* (9.94%), *Apodemus agrarius* (8.43%), and *Rattus losea* (1.51%). Other species included *Niviventer fulvescens, Rattus nitidus, Niviventer confucianus, Microtus fortis* Buchner, *Leopoldamys edwardsi, Berylmys bowersi, Eothenomys melanogaster, Micromys minutus*, and *Apodemus draco* (11.75%). The highest and lowest densities were found in Wenzhou and Hangzhou and Ningbo, respectively.

In 2020, 15,719 m of grassland and 261 animals were investigated at six monitoring sites; 124 dissociate and 252 ectoparasitic ticks were caught, with a dissociate tick density of 0.79 and ectoparasitic tick density of 0.97. Dissociate ticks were only found in Ningbo and Lishui, whereas ectoparasitic ticks were collected in Ningbo, Yiwu, Lishui, and Taizhou. In Hangzhou and Quzhou, no dissociate or ectoparasitic ticks were found. Six species were identified, the most abundant being *Haemaphysalis longicornis* (56.38%), followed by *Rhipicephalus sanguineus* (33.51%), *Rhipicephalus haemaphysaloides* (7.98%), *Ixodes sinensis* (1.06%), and others (such as *Ixodes granulatus* and *Rhipicephalus microplus* [1.07%]).

In 2020, 903 chigger mites were detected on 64 rodents at two monitoring sites, with a density of 14.11 (Table 3). Among all chigger mites detected, 218 samples were identified. The most abundant species was *Walchia* spp. mite (68.35%), followed by *Leptotrombidium deliense* (31.65%). Two chigger mites were collected from Yiwu and Lishui.

### Pathogen surveillance

In total, 1,345 rodents were sampled and tested for bacterial and viral pathogens. All 1,345 rodents were tested for hantavirus, SFTSV, and pathogenic *Leptospira* spp., and the positivity rates of SFTSV was 0.00%, while the positivity rates of hantavirus and pathogenic *Leptospira* spp. were 1.86 and 7.36%, respectively (Table 4). *O. tsutsugamushi* was tested in 853 rodents, with a positivity rate of 0.35%. *Yersinia* spp. and *R. mordeni* were tested in 229 and 107 rodents, with a null positivity rate for both. *Bartonella* spp. was tested in 156 rodents, with a positivity rate of 7.05%. Hantavirus was mainly found in *R. losea* (2.67%), *A. agrarius* (2.47%), *R. norvegicus* (2.43%), *R. flavipectus* (2.99%), and *M. fortis* (5.26%) in the Zhejiang Province, and The pathogenic bacteria *Leptospira* spp. was mainly found in *M. fortis* (31.58%), *E. melanogaster* (16.36%) and *R. losea* (12.00%). *O. tsutsugamushi* was only found in *R. losea* (2.90%) and *R. flavipectus* (0.83%), and *Bartonella* spp. was mainly found in *A. agrarius* (21.95%) and *N. confucianus* (16.67%).

**Table 4:** The prevalence of pathogen infections in different rodent species in Zhejiang Province, 2020.

**Sites/Rodent species**	***Hantavirus***		***SFTS*** **virus**		**The pathogenic bacteria** ***Leptospira*** **spp**.		***Orientia tsutsugamushi***		***Yersinia*** **spp**.		**Rickettsia mordeni**		***Bartonella*** **spp**.	
	**No. of rodents**	**Positive rate (%)**	**No. of rodents**	**Positive rate (%)**	**No. of rodents**	**Positive rate (%)**	**No. of rodents**	**Positive rate (%)**	**No. of rodents**	**Positive rate (%)**	**No. of rodents**	**Positive rate (%)**	**No. of rodents**	**Positive rate (%)**
*Rattus losea*	75	2.67	75	0.00	75	12.00	69	2.90	6	0.00	-	-	-	-
*Apodemus agrarius*	283	2.47	283	0.00	283	11.31	175	0.00	29	0.00	38	0.00	41	21.95
*Niviventer fulvescens*	14	0.00	14	0.00	14	0.00	7	0.00	-	-	7	0.00	-	-
*Rattus norvegicus*	329	2.43	329	0.00	329	9.42	204	0.00	72	0.00	18	0.00	35	2.86
*Suneus murinus*	151	0.00	151	0.00	151	1.32	93	0.00	48	0.00	6	0.00	4	0.00
*Rattus flavipectus*	234	2.99	234	0.00	234	2.14	121	0.83	33	0.00	32	0.00	48	0.00
*Mus musculus*	113	0.00	113	0.00	113	0.88	110	0.00	2	0.00	1	0.00	-	-
*Niviventer confucianus*	53	0.00	53	0.00	53	7.55	35	0.00	8	0.00	4	0.00	6	16.67
*Rattus nitidus*	1	0.00	1	0.00	1	0.00	-	-	-	-	1	0.00	-	-
*Leopoldamys edwardsi*	15	0.00	15	0.00	15	0.00	15	0.00	-	-	-	-	-	-
*Apodemus draco*	3	0.00	3	0.00	3	0.00	3	0.00	-	-	-	-	-	-
*Microtus fortis Buchner*	19	5.26	19	0.00	19	31.58	19	0.00	-	-	-	-	-	-
*Eothenomys melanogaster*	55	0.00	55	0.00	55	16.36	2	0.00	31	0.00	-	-	22	0.00
**Total**	1345	1.86	1345	0.00	1345	7.36	853	0.35	229	0.00	107	0.00	156	7.05

In 2020, a total of 10,200 female mosquitoes were tested for disease borne pathogens, including *Ae. albopictus* (*n* = 5,000), *Cx. pipiens pallens* (*n* = 3,000), and *Cx. tritaeniorhynchus* (*n* = 2,200). No flavivirus or alphavirus was found in Zhejiang Province.

### Insecticide resistance surveillance in *Ae. Albopictus*

Insecticide susceptibility tests were conducted on field populations from five monitoring sites. Except for the Shaoxing population, none of the other field populations of *Ae. albopictus* were sensitive to the pyrethroids tested (Table 5A). The 24 h mortality rates ranged from 52.00 to 89.39%, 7.14 to 80.30%, 33.33 to 86.57%, and 60.49 to 90.41% after exposure to 0.07% lambda-cyhalothrin, 0.08% beta-cypermethrin, 0.03% deltamethrin, and 0.4% permethrin, respectively. Hangzhou showed the highest resistance to all four insecticides tested.

**Table 5:** Insecticide resistance of *Aedes albopictus* against pyrethroids, organophosphorus, and carbamate.

**(5A)**								
**Sites**	**0.07% Lambda- cyhalothrin**		**0.08% Beta-cypermethrin**		**0.03% Deltamethrin**		**0.4% Permethrin**	
	**24h-mortality(%)**	**Resistance status**	**24h-mortality(%)**	**Resistance status**	**24h-mortality(%)**	**Resistance status**	**24h-mortality(%)**	**Resistance status**
Hangzhou	52.00	resitance	7.14	resitance	33.33	resitance	60.49	resitance
Ningbo	-	-	8.54	resitance	49.25	resitance	66.10	resitance
Shaoxing	-	-	-	-	100.00	susceptibility	100.00	susceptibility
Quzhou	-	-	-	-	86.57	resitance	90.41	probable resistance
Yiwu	89.39%	resitance	80.30	resitance	60.00	resitance	78.67	resitance
**(5B)**										
**Sites**	**Organophosphorus**						**Carbamate**			
	**0.5% Malathion**		**0.2% Fenitrothion**		**2% Chlorpyrifos**		**0.05% Propoxur**		**0.2% Bendiocarb**	
	24 h-mortality(%)	Resistance status	24 h-mortality(%)	Resistance status	24 h-mortality(%)	Resistance status	24 h-mortality(%)	Resistance status	24 h-mortality(%)	Resistance status
Hangzhou	100.00	susceptibility	100.00	susceptibility	-	-	100.00	susceptibility	100.00	susceptibility
Ningbo	92.98	probable resistance	-	-	100.00	susceptibility	100.00	susceptibility	-	-
Shaoxing	96.67	probable resistance	-	-	-	-	100.00	susceptibility	-	-
Quzhou	84.91	resistance	88.68	resistance	-	-	92.45	probable resistance	-	-
Yiwu	98.73	susceptibility	100.00	susceptibility	100.00	susceptibility	100.00	susceptibility	100.00	susceptibility

Five *Ae. albopictus* field populations showed different resistance levels to 0.5% malathion, with the 24 h mortality rate ranging from 84.91 to 100.00% (Table 5B). The Hangzhou and Yiwu populations were susceptible after exposure to 0.5% malathion, the Ningbo and Shaoxing populations showed probable resistance, and the Quzhou population showed resistance. Two (Hangzhou and Yiwu populations) out of the three populations exposed to 0.2% fenitrothion showed susceptibility, and the rest of the population in Quzhou showed resistance, with a 24 h mortality rate of 88.68%. Both populations in Ningbo and Yiwu showed susceptibility after exposure to 2% chlorpyrifos. Hangzhou and Yiwu populations showed susceptibility to all organophosphorus insecticides tested whereas the Quzhou population was resistant to these insecticides. With respect to carbamate, four out of the five field populations were susceptible to 0.05% propoxur, whereas the rest of the populations in Quzhou had probable resistance, with a 24 h mortality rate of 92.45%. Two populations tested from Hangzhou and Yiwu were all susceptible to 0.2% bendiocarb.

## Discussion

This study monitored the ecology of mosquitoes, rodents, ticks, and chigger mites, etiology of mosquitoes and rodents, and drug resistance for mosquitoes (*Ae. albopictus*). Knowledge of vector populations, pathogen infections, and drug resistance in this study may provide important information for early detection and control of VBDs. According to the present study, mosquitoes, rodents, ticks, and chigger mites were widely distributed in Zhejiang Province, with varying populations in different monitoring sites. Although no flavivirus or alphavirus was found in the mosquitoes sampled, hantavirus, the pathogenic bacteria *Leptospira* spp., *O. tsutsugamushi*, and *Bartonella* spp. were found in the rodents collected. *Ae. albopictus* was widely resistant to pyrethroids and should be used cautiously in the future for mosquito control.

Four mosquito species were identified from samples collected *via* CDC light traps in this study; the dominant species being *Cx. tritaeniorhynchus*. *Ae. albopictus* was found in only 1.26% of the total captured adult mosquitoes. This could be attributed to the fact that CDC light traps mainly collected nocturnal or crepuscular mosquito species, and diurnal mosquitoes, such as *Ae. albopictus*, could have been underestimated. To compensate for this defect, we conducted larval mosquito surveillance to monitor *Aedes* mosquitoes; *Ae. albopictus* was distributed across all sites monitored in Zhejiang Province. Moreover, its density was significantly high (BI > 20), and half of the monitoring sites had a risk of dengue fever (26). In this study, no flavivirus or alphavirus was found. This may be caused by coronavirus disease 2019 (COVID-19) (27). Due to the measures to prevent COVID-19, the prevalence of VBDs in 2020 have decreased by 55% compared to that in 2015–2019 (27) in Zhejiang province. That is because most MBD cases in the Zhejiang Province were imported. Similarly, international mobility restriction decreased the incidence rate of some MBDs, such as indigenous cases of dengue fever, in the Zhejiang Province to zero in 2020 and 2021 (unpublished data). However, in 2018, dengue virus genotype I was detected in *Ae. albopictus* sampled in Wenzhou during a local dengue fever outbreak (28). Thus, under normal travel conditions, the prevalence of flavivirus or alphavirus might increase. According to the results of insecticide susceptibility tests, almost all *Ae. albopictus* field populations showed different degrees of resistance to pyrethroids. This suggests that pyrethroids, known to be highly efficient and less toxic to mammals, will no longer be applicable to mosquito control in the near future in the Zhejiang Province. Accordingly, we infer that the risk of MBDs was low at present because none of the pathogens were detected, and imported cases were restricted. However, the main mosquito populations monitored, such as *Cx. tritaeniorhynchus, Cx. pipiens pallens*, and *Ae. albopictus*, have high vector competence, and their densities were high. When MBDs outbreaks and mosquito control are urgently required, organophosphorus and carbamate insecticides could be rotated to better manage adult indoor and outdoor mosquitoes.

Rodents play important roles in the transmission and epidemiology of various VBDs, such as plague, hemorrhagic fever, and leptospirosis, as reservoir hosts. Many studies have demonstrated that the pathogen infection rates of hantavirus and *Leptospira* spp. species in rodents are positively correlated with the incidence rates of hemorrhagic fever and leptospirosis in the population, which indicates the importance of monitoring rodents for the prediction, prevention, and control of VBDs (29, 30). According to our results, except for SFTSV, *Yersinia* spp., and *R. mordeni*, four out of the seven pathogens tested were detected in rodents in Zhejiang Province, among which *Leptospira* spp. and *Bartonella* spp. were the most prevalent. Both bacterial species mainly circulated in *A. agrarius*, with infection rates of 11.31% and 21.95%, respectively. *A. agrarius* mice were significantly common in Zhejiang, accounting for 8.43% of all rodents caught. In contrast, *A. agrarius* is one of the two rodent hosts (*R. norvegicus* and *A. agrarius*) that transmit hantavirus in China. The hantavirus infection rate of *A. agrarius* was 2.47%. Therefore, *A. agrarius* should be the key rodent species for monitoring and control in Zhejiang Province.

Ticks and chigger mites were found in 66.67% (4/6) and 100% (2/2) of all monitoring sites, respectively, suggesting that ticks and chigger mites are widespread in Zhejiang Province. Accordingly, six tick species and two chigger mite species were identified, with the most abundant species being *H. longicornis* (56.38%) and *Walchia* spp. mite (68.35%). *H. longicornis* is the main vector transmitting SFTSV in China. SFTS is a natural focal disease with a 30% early mortality rate (31). The seasonal density of dissociate ticks is positively related to the number of SFTS cases (32). Zhejiang Province is one of the seven main SFTS natural foci in China and dozens of SFTS cases have been reported annually (33). Regular monitoring and control of ticks have been proposed as important methods to prevent SFTS.

In this integrated surveillance exercise, ecology and etiology monitoring, and drug resistance monitoring were connected. Through mosquito ecology monitoring, we obtained mosquitoes required to survey the prevalence of various pathogens and sensitivity against multiple insecticides. Rodents were used to monitor chigger mites and samples to test rodent-derived bacterial and viral pathogens. Repetitive work was restricted. According to our previous research, integrated surveillance of rodents could save 21.6% of the cost compared to traditional rodent surveillance systems (34). The limitation to the study was that the pathogen survey was only conducted in mosquitoes and rodents. In the near future, we will test pathogens in other vectors to gain a comprehensive knowledge of vectors and VBDs in Zhejiang Province.

In conclusion, integrated surveillance systems of multiple arthropod vectors (mosquitoes, ticks, mites) and animal reservoirs (rodents) in 2020 could provide invaluable information for the management and control of VBD epidemics, saving both human lives and financial resources.

## Data availability statement

The original contributions presented in the study are included in the article/supplementary material, further inquiries can be directed to the corresponding author.

## Author contributions

YYW manuscript writing and data analysis. JNW, QML, TQL, and MYL data collection. ZYG project design. All authors contributed to the article and approved the submitted version.

## Funding

This work was supported by the Zhejiang Medical and Health Science and Technology plan (No. 2022KY720).

## Conflict of interest

The authors declare that the research was conducted in the absence of any commercial or financial relationships that could be construed as a potential conflict of interest.

## Publisher's note

All claims expressed in this article are solely those of the authors and do not necessarily represent those of their affiliated organizations, or those of the publisher, the editors and the reviewers. Any product that may be evaluated in this article, or claim that may be made by its manufacturer, is not guaranteed or endorsed by the publisher.

## Abbreviations

CDC: Centers for Disease Control and Prevention
BI: Breteau index
MBD: Mosquito-borne diseases
PCR: Polymerase chain reaction
SFTSV: Severe fever with thrombocytopenia syndrome bunia virus
VBD: Vector-borne diseases
WHO: World Health Organization.

## References

[1] WHO. Global Vector Control Response 2017-2030. Geneva: WHO (2017).

[2] Liu QY. Epidemic profile of vector-borne diseases and vector control strategies in the new era (review). Chin J Vector Biol Control. (2019) 30:1–11. 10.11853/j.issn.1003.8280.2019.01.001

[3] Liu QY. Impact of climate change on vector-borne diseases and related response strategies in China: major research findings and recommendations for future research. Chin J Vector Biol Control. (2021) 32:1–11. 10.11853/j.issn.1003.8280.2021.01.001

[4] Wu HX Liu XB Liu QY. Current situation and problems of vector control in China Capital. J Public Health. (2018) 12:1–6. 10.16760/j.cnki.sdggws.2018.01.002

[5] Cheng Q Jing QL Robert CS Marshall JM Yang ZC Gong P. Climate changes of China's mainland over the past half century. Acta Meteor Sin. (2005) 63:942–56. 10.3321/j.issn:0577-6619.2005.06.011

[6] Wu F Liu Q Lu L Wang J Song X Ren D. Distribution of Aedes albopictus(Diptera:Culicidae) in northwestern China. Vector BorneZoonotic Dis. (2011) 11:1181–86. 10.1089/vbz.2010.0032 21254912

[7] Lai HB Luo LK Ou QH Fen ZL Zhang H He ZQ. Analysis on correlation between the density of dengue fever vector and climatic factors in Yunfu city. Chin J Hyg Insect Equip. (2018) 2451–55. 10.19821/j.1671-2781.2018.01.016

[8] Ge WX Jin KK Sun LN Liu QY Yin LP Li J. Research advances in the relationship between dengue epidemic and different meteorological factors. Chin J Vector Biol Control. (2019) 30:367–70. 10.11853/j.issn.1003.8280.2019.04.002

[9] Yu X. Comparative Study on Tolerance Range of Aedes albopictus and Ae. Aegypti to Temperature and Photoperiod. Beijing: Chinese Center for Disease Control and Prevention. (2013).

[10] Li S Tao HY Xu Y. Abiotic determinants to the spatial dynamics of dengue fever in Guangzhou Asia. Pac J Public Health. (2013) 25:239–47. 10.1177/1010539511418819 21852418

[11] Wu YY Wang JN TQ Li QM Liu ZY Gong Hou J. Effect of different carbon dioxide (CO2) flows on trapping Aedes albopictus with BG traps in the field in Zhejiang Province, China. PLoS ONE. (2020) 15:e0243061. 10.1371/journal.pone.0243061 33259534 PMC7707600

[12] Ramírez AL van den Hurk AF Meyer DB Ritchie SA. Ritchie. Searching for the proverbial needle in a haystack: advances in mosquito-borne arbovirus surveillance. Parasit Vectors. (2018) 11:320. 10.1186/s13071-018-2901-x 29843778 PMC5975710

[13] A Pautasso R Desiato S Bertolini N Vitale M C Radaelli M Mancini. Mosquito surveillance in Northwestern Italy to monitor the occurrence of tropical vector-borne diseases. Transbound Emerg Dis. (2013) 60 (Suppl. 2):154–61. 10.1111/tbed.12123 24589116

[14] Lerdthusnee JNi. T Monkanna W Leepitakrat S Leepitakrat S Insuan. Surveys of rodent-borne disease in Thailand with a focus on scrub typhus assessment. Integrative Zoology. (2008) 3:267–73. 10.1111/j.1749-4877.2008.00100.x 21396076

[15] P Mulatti L Bonfanti G Capelli K Capello M Lorenzetto C Terregino. West Nile virus in north-eastern Italy, 2011: entomological and equine IgM-based surveillance to detect active virus circulation. Zoonoses Public Health. (2013) 60:375–82. 10.1111/zph.12013 22971022

[16] Regis L Souza WV. AF Furtado CD Fonseca JC Silveira Jr PJ Ribeiro Jr. An entomological surveillance system based on open spatial information for participative dengue control Anais da. Academia Brasileira de Ciencias. (2009) 81:655–62. 10.1590/S0001-37652009000400004 19893891

[17] Engler G Savini A Papa J Figuerola M Groschup H Kampen H. European Surveillance for West Nile Virus in Mosquito Populations. Int J Environ Res Public Health. (2013) 10:4869-95. 10.3390/ijerph10104869 24157510 PMC3823308

[18] Wang Z Ling F Liu Y Ren JP Sun JM. Epidemiological characteristics of dengue fever in Zhejiang province, China, 2015-2019. J Dis Sur. (2020) 31:643–7. 10.11853/j.issn.1003.8280.2020.06.003

[19] China National Standardization Administration Committee Surveillance methods for vector density-Mosquito (GB/T 23797-2009). Available online at: https://www.doc88.com/p-7337313094912.html

[20] China National Standardization Administration Committee Surveillance methods for vector density-Rodent (GB/T 23798-2009). Available online at: https://www.doc88.com/p-5999909846407.html

[21] Pang Z. Establishment of multiplex real time PT-PCR assays for hemorrhagic fever virus detection. Chinese Center for Disease Control and Prevention. (2013).

[22] Sun YL Liang MF. J Qu C Jin QF Zhang JD Li. Early diagnosis of novel SFTS bunyavirus infection by quantitative realtime RT-PCR assay. Clin Virol. (2012) 53:48–53. 10.1016/j.jcv.2011.09.031 22024488

[23] Wei Y Zhou Y Wang CH Zhao SH Zhang QW Wan CS. Rapid detection of yersina pestis with Taqman probe by real time fluorescent quantitave PCR. Chin J Control Prev. (2012) 16:954–7.

[24] WHO. Monitoring and Managing Insecticide Resistance in Aedes Mosquito Populations. Geneva: World Health Organization. (2016).

[25] National Health Commission of the People's Republic of China. National Vector Surveillance Program. Beijing: China Center for Disease Control and prevention (2016).

[26] Li XN Luo L. XC Xiao QL Jing YH Wei YL Li. Using Breteau Index to analyze thenature of sporadic and outbreak cases of Dengue fever. Chin J Epidemiol. (2014) 35:821–4. 25294075

[27] Ding ZY Wu HC Lu QB Wu C Lin JF. Epidemiology characteristics of the notuficable infectious diseases reported in zhejiang province, 2020. Prev Med. (2021) 33:325–30.

[28] QM Liu JN Wang J Hou YY Wu HD Zhang D Xing. The Investigation of Aedes (Stegomyia) albopictus and Detection of Dengue Virus Type 1 in the Species during the 2018–2020 Outbreak in Zhejiang Province, China. Front Cell Infect Microbiol. (2022) 12: 834766. 10.3389/fcimb.2022.834766 35846756 PMC9283783

[29] Wu T Mao LL. Correlation between hemorrhagic fever with renal syndrome and rodent virus index in Jinzhou. Chin J Hyg Insect Equip. (2018) 24:71–3. 10.19821/j.1671-2781.2018.01.022

[30] Xu WJ Zhang K. Relationship between seasonal fluctuation and epidemic situation of *Leptospira* carried by wild rats. Chinese J Zoonoses. (1994) 10:55–6.

[31] XJ Yu MF Liang SY Zhang Y Liu JD Li YL Sun. Fever with thrombocytopenia associated with a novel bunyavirus in China. N Engl J Med. (2011) 364:1523–32. 10.1056/NEJMoa1010095 21410387 PMC3113718

[32] Lei XJ Kong JP Xiong JF Tan LF. Epidemiological characteristics of severe fever with thrombocytopenia syndrome and association with tick density in Chongyang County, Hubei Province. Shanghai J Prevent Med. (2022). (in press).

[33] Zhang QT Sun JM Ling F Shi XG Ren JP Guo S. Analysis of reported cases of fever with thrombocytopenia syndrome andtick vectors surveillance results in Zhejiang province of China in 2021. Chin J Vector Biol Control. (2022) 33:485–8. 10.11853/j.issn.1003.8280.2022.04.008

[34] Wu YY Ling F. J Hou, S Guo, JN Wang, ZY Gong. Will integrated surveillance systems for vectors and vector-borne diseases be the future of controlling vector-borne diseases? A practical example from China(review). Epidemiol Infect. (2016) 144:1895–903. 10.1017/S0950268816000297 26899818 PMC9150650

